# Progressive myalgia as the sole manifestation of cancer-associated myositis: A case report and review of the literature

**DOI:** 10.1097/MD.0000000000046170

**Published:** 2025-11-14

**Authors:** Jian Xu, Shucong Peng, Xiuqing Huang

**Affiliations:** a Department of Rehabilitation, The Quzhou Affiliated Hospital of Wenzhou Medical University, Quzhou People’s Hospital, Quzhou, China; b Department of Rehabilitation Medicine, Huashan Hospital, Fudan University, Shanghai, China.

**Keywords:** anti-PL-7 antibody, anti-TIF1γ antibody, cancer-associated myositis, colon cancer, idiopathic inflammatory myopathies

## Abstract

**Rationale:**

Cancer-associated myositis (CAM) is a rare autoimmune disease linked to underlying malignancies and classified as an idiopathic inflammatory myopathy. It is characterized by a complex pathogenesis, poor prognosis, and diverse symptoms such as muscle weakness and myalgia, which may be accompanied by extramuscular manifestations. The presence of comorbidities in elderly patients can mask these symptoms, posing significant diagnostic and treatment challenges. Early recognition of CAM and detection of malignancy are crucial for improving patient outcomes.

**Patient concerns:**

A 71-year-old male presented with progressive symmetrical proximal muscle pain as the sole manifestation, without typical extramuscular manifestations. Initial tests revealed negative muscle enzyme results, but electromyography indicated myogenic damage in the proximal muscles of the limbs. Further serological testing detected positive anti-TIF1γ and anti-PL-7 antibodies.

**Diagnoses:**

The patient was diagnosed with CAM based on the presence of specific autoantibodies and a rapid clinical response to corticosteroids.

**Interventions:**

Treatment with low-dose hormone therapy resulted in significant improvement of symptoms. A comprehensive malignancy screening was performed.

**Outcomes:**

Subsequent surgical pathology confirmed the presence of colon cancer. The patient’s condition improved markedly with timely intervention and targeted treatment. At 6-month follow-up, he remained symptom-free.

**Lessons:**

This case underscores the importance of considering CAM in patients with unexplained symmetrical proximal muscle pain, even in the absence of typical extramuscular symptoms. The detection of myositis-specific autoantibodies is critical for accurate diagnosis. Early identification and treatment of the underlying malignancy can significantly improve prognosis. Future research should focus on elucidating the pathogenesis of CAM, identifying more specific biomarkers, and developing standardized diagnostic and therapeutic guidelines to enhance early diagnosis and treatment outcomes.

## 1. Introduction

Cancer-associated myositis (CAM) is a rare autoimmune disease usually associated with an underlying malignancy. It belongs to the group of idiopathic inflammatory myopathies (IIMs), but compared to other types of myositis, such as polymyositis and dermatomyositis, CAM has a more complex pathogenesis and poorer prognosis.^[1]^ The clinical manifestations of IIMs vary, with muscle weakness and myalgia being common symptoms, but they often also involve extramuscular manifestations, such as rash, arthritis, interstitial lung disease, and cardiac involvement. In some patients, extramuscular manifestations may dominate the clinical presentation, with no muscle weakness, making diagnosis challenging.^[2]^ In CAM, symptoms may be masked by the underlying tumor or associated with tumor treatment, especially in elderly patients who often have multiple underlying diseases and reduced immune system function, making the clinical manifestations of CAM even more insidious and prone to misdiagnosis or underdiagnosis. Additionally, elderly patients are less tolerant to treatment, further increasing the difficulty of treatment. Therefore, early recognition of CAM and timely detection of potentially malignant tumors are crucial for improving patient prognosis.

## 2. Case presentation

A 71-year-old male, weighing 61 kg, with no family history of genetic disease, presented with a 3-month history of progressive pain in both upper arms without any obvious precipitating factors. The pain was persistent and distending in nature. Initially, it was tolerable and not associated with numbness, weakness of the upper limbs, or fever. During this time, the patient sought treatment at local hospitals on several occasions and underwent acupuncture, local physiotherapy, and other conservative therapies, but his pain did not improve and gradually worsened. Three days before admission, the pain in both upper arms intensified, rendering him unable to lift his upper limbs. This was accompanied by new-onset pain extending from the posterior aspects of both knees to the medial thighs, which made walking impossible. The maximum pain intensity reached 8 on the numerical rating scale. On admission, physical examination revealed no enlarged lymph nodes, skin rash, or significant muscle tenderness in the proximal limbs. Muscle strength assessment was limited due to severe pain, and pathological reflexes were not elicited. Laboratory tests revealed mild anemia (hemoglobin 117 g/L) and elevated ultrasensitive C-reactive protein (82.53 mg/L). Myoenzyme profile, tumor markers, and antinuclear antibody panel were unremarkable. Electromyography (EMG) demonstrated myogenic damage in the proximal muscles of both upper and lower limbs. Cervical spine magnetic resonance imaging and chest computed tomography revealed no abnormalities.

**Figure 1.**
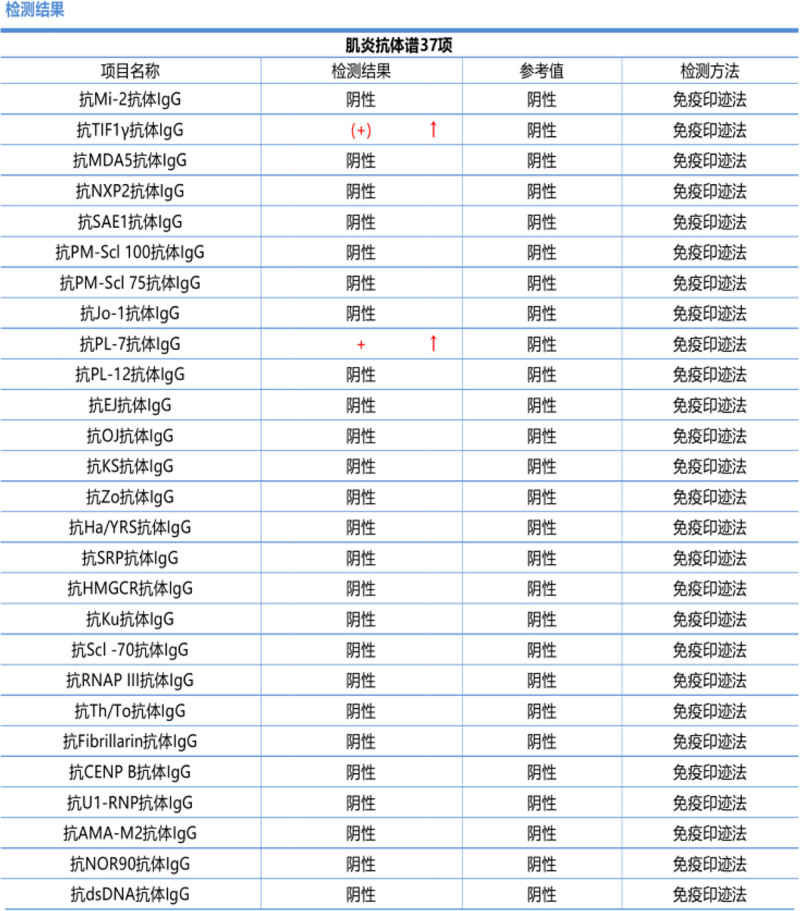
Myositis antibody profile indicated: anti-TIF1γ antibody IgG (+), anti-PL-7 antibody IgG (+).

**Figure 2.**
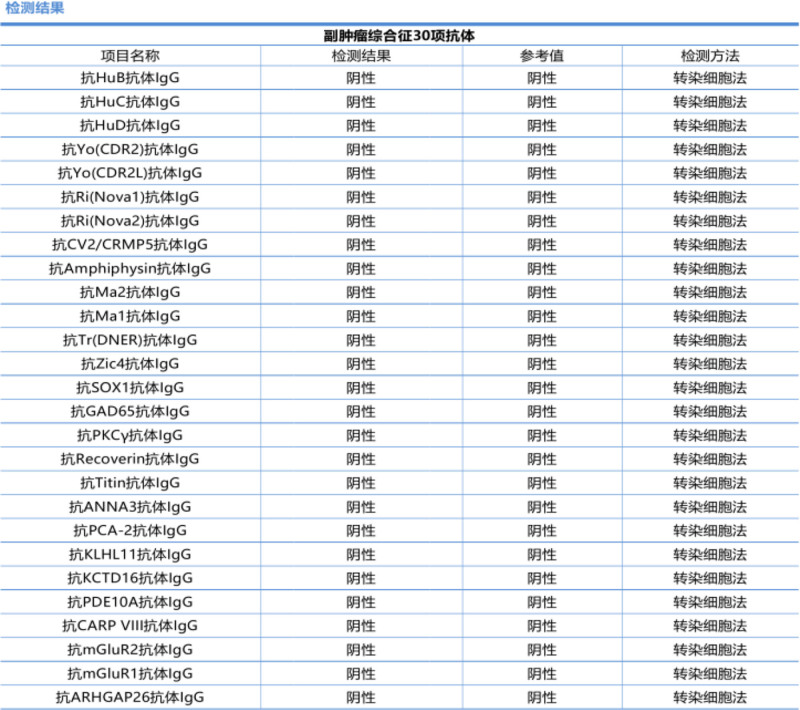
Colonoscopy paraneoplastic-related antibodies showed no significant abnormalities.

**Figure 3.**
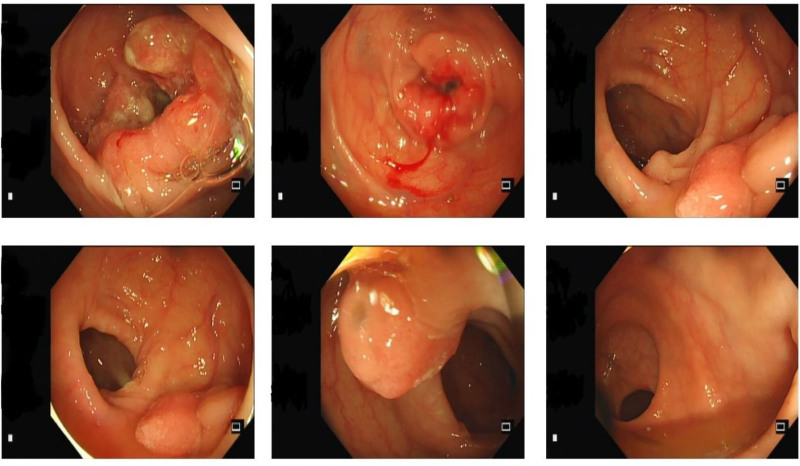
Colonoscopy.

**Figure 4.**
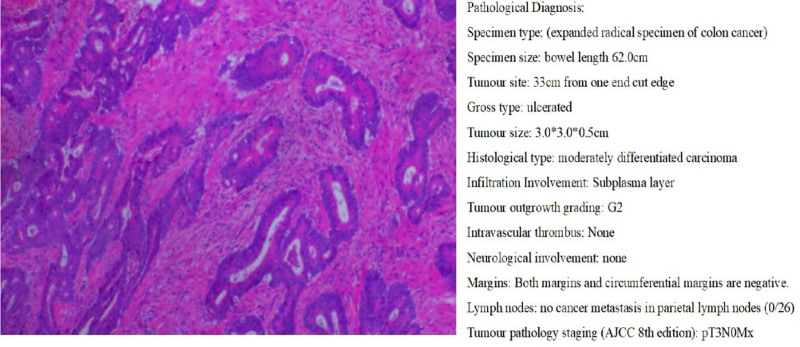
Postoperative pathology of colon cancer.

**Figure 5.**
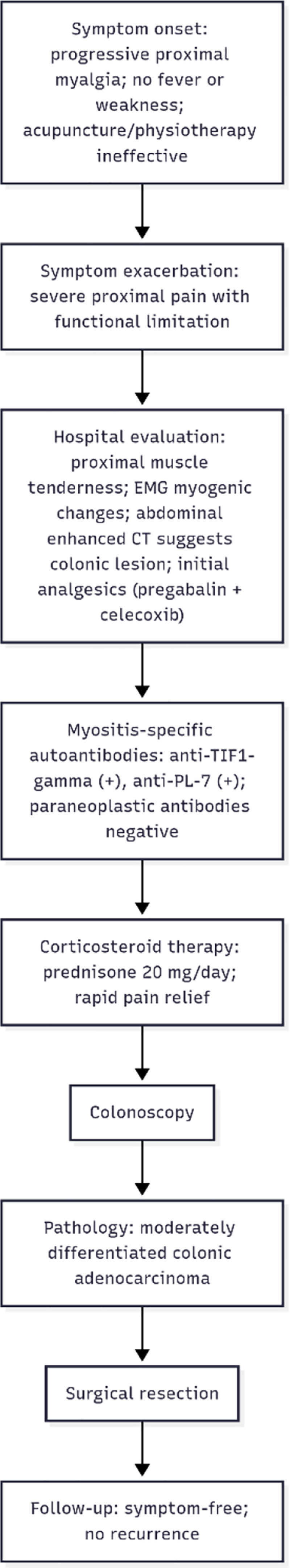
Schematic diagnostic pathway of the present case.

Initial pain control with oral pregabalin capsules and celecoxib capsules provided minimal relief. A myositis-specific antibody panel subsequently returned positive for anti-TIF1γ antibody IgG (+) and anti-PL-7 antibody IgG (+) (Fig. 1), while paraneoplastic antibodies were negative (Fig. 2). An enhanced computed tomography scan of the entire abdomen revealed localized thickening of the bowel wall at the splenic flexure of the colon, prompting colonoscopy, which identified descending colon cancer and multiple colonic polyps (Fig. 3). Given the diagnosis of CAM, oral prednisolone was initiated at a total daily dose of 20 mg (10 mg twice daily). Three days later, the patient’s pain in both upper arms and knees improved significantly, with the numerical rating scale score decreasing to 2. He regained the ability to lift both upper limbs independently and walk without assistance. The patient subsequently underwent surgical resection in the Department of Surgery. Postoperative histopathology confirmed moderately differentiated adenocarcinoma of the colon (Fig. 4). No adverse events occurred during diagnosis and treatment. At a 6-month follow-up, the patient reported complete resolution of myalgia, full recovery of limb function, and no evidence of cancer recurrence on surveillance imaging and laboratory testing. The patient’s clinical course is summarized in Table 1. The stepwise diagnostic reasoning is summarized in a schematic flow diagram (Fig. 5), corresponding to the events listed in Table 1.

**Table 1:** Timeline of case events.

Date/time	Event/clinical course	Key findings/actions	Outcome
~Month ‐3	Symptom onset	Persistent, distending pain in both upper arms, no fever, numbness, or weakness; local acupuncture/physiotherapy ineffective	Symptoms gradually worsened
~3 days before admission	Symptom exacerbation	Severe bilateral upper arm pain; new pain behind knees radiating to medial thighs; unable to lift arms or walk; NRS = 8	Functional disability
Day 1–3 (admission)	Hospital admission	Physical exam: obvious proximal limb muscle tenderness, no rash, no lymphadenopathy; labs: mild anemia, ↑CRP; myoenzyme and tumor markers normal; EMG: myogenic damage (proximal upper and lower limbs); cervical MRI and chest CT unremarkable; abdominal enhanced CT: localized thickening at splenic flexure of colon → colonoscopy recommended	Diagnosis work-up initiated
Day 1–4	Initial pain control	Oral pregabalin + celecoxib	Pain persisted
Day 4	Myositis antibody testing/paraneoplastic Ab	Anti-TIF1γ IgG (+), anti-PL-7 IgG (+); paraneoplastic Ab negative	Suspected cancer-associated myositis (CAM)
Day 5	Corticosteroid therapy initiated	Oral prednisone 10 mg twice daily (20 mg/d)	Marked pain relief within 3 days; NRS ↓ to 2; regained ability to lift arms and walk
Day 9	Colonoscopy	Descending colon cancer + multiple colon polyps	Prepared for surgery
Day 22	Surgery	Resection; histopathology: moderately differentiated adenocarcinoma	No perioperative complications
Month +6	Follow-up	No recurrence; symptom-free	Maintained remission

CRP = C-reactive protein, CT = computed tomography, MRI = magnetic resonance imaging, NRS = numerical rating scale.

Patient perspective:

“When the pain in my arms and legs became worse and I could no longer walk, I felt anxious and uncertain about what was happening to me. Receiving a clear diagnosis helped me understand my condition, and I was relieved when the treatment worked so quickly. Although the surgery was challenging, I am grateful that the underlying problem was identified and treated in time.”

## 3. Discussion

Currently, there are no unified guidelines for the subclassification of IIMs, which typically include dermatomyositis, polymyositis, anti-synthetase syndrome, inclusion body myositis, and immune-mediated necrotizing myopathy.^[3–5]^ Although there have been reports of associations between cancer and myositis more than 100 years ago, there are still no specific diagnostic criteria for CAM subtypes, which may be related to the limited number of reported CAM cases and diversity of clinical presentations. Some scholars also suggest that the term “cancer-associated myositis” refers to IIMs forms observed within 3 years after the appearance of malignant tumor characteristics.^[6,7]^ Patients with IIM often present with subacute onset of symmetrical proximal muscle weakness, elevated muscle enzymes, EMG showing myopathic changes, and abnormal autoantibodies.^[8,9]^ In this case, the patient’s main symptom was symmetrical proximal muscle pain, and EMG suggested myogenic damage, but there were no abnormalities in the muscle enzyme profile, which complicated the diagnosis. Pain is generally caused by muscle inflammation and muscle fiber damage, especially in CAM, where the inflammatory response may be more pronounced. The symmetrical and proximal distribution of pain suggests the possibility of inflammatory myopathy, and this pain pattern provides an important diagnostic clue. IIMs are often accompanied by typical extramuscular manifestations such as skin changes, arthritis, and interstitial lung disease,^[9,10]^ this case lacked the above extramuscular manifestations, indicating a lack of specific clinical manifestations in the pathogenesis of CAM. The lack of extramuscular manifestations such as skin changes or arthritis further highlights the heterogeneous and sometimes subtle nature of CAM.

A significant advancement in the field of myositis has been the discovery of myositis-specific autoantibodies (MSAs), which are present in up to 60% of IIMs patients. MSAs are closely associated with different clinical phenotypes, making them important for predicting organ involvement and possibly prognosis.^[2,11,12]^ In our patient, the positive results for anti-TIF1γ and anti-PL-7 antibodies provided crucial evidence for the diagnosis of CAM. Anti-TIF1γ antibody is a sensitive and specific serological marker for dermatomyositis associated with tumors, and its positive result suggests the possibility of underlying malignant tumors.^[13–15]^ Anti-PL-7 antibody belongs to a group of autoantibodies against aminoacyl-tRNA synthetase and is also frequently detected in CAM, with its positive result possibly linked to tumors accompanied by interstitial lung disease, serving as a valuable predictor for patients with myositis and tumors.^[16–18]^ In this case, the patient tested negative for paraneoplastic syndrome-related antibodies, while anti-TIF1γ and anti-PL-7 antibodies were positive, which is not contradictory because anti-TIF1γ and anti-PL-7 antibodies are primarily associated with CAM, whereas paraneoplastic autoantibodies (such as CV2, amphiphysin, Ma2, Ri, Hu, and Yo) are usually associated with neurologic paraneoplastic syndromes.^[19–21]^ These 2 types of antibodies target different antigens and may be involved in distinct immune mechanisms. Therefore, a negative result for paraneoplastic syndrome-related antibodies does not rule out the possibility of CAM. Although studies have indicated a specific association between anti-TIF1γ antibodies and colon cancer,^[22]^ no specific myositis antibody marker has been definitively shown to have a direct connection with colon cancer. The co-positivity of anti-TIF1γ and anti-PL-7 is uncommon. While anti-TIF1γ has a robust association with malignancy, anti-PL-7 is a hallmark of the anti-synthetase syndrome. Their coexistence in our patient may suggest a complex immunological overlap or a unique pathogenic mechanism. This rare dual positivity further reinforces the need for a comprehensive malignancy search when such antibody profiles are encountered, as their combined presence could signify a particularly high risk of underlying cancer.^[23–26]^ This case suggests that the presence of anti-TIF1γ and anti-PL-7 antibodies may have implications for the diagnosis of rectal cancer.

In the treatment of CAM, it is usually necessary to comprehensively consider the severity of illness, age, comorbidities, and the presence or absence of underlying tumors. Treatment of IIMs typically involves 1 or more immunomodulators.^[3]^ UK guidelines recommend oral prednisolone for adults at a dose of 0.5 to 1 mg/kg/d, usually 40 to 60 mg.^[27]^ The choice of a lower initial dose of prednisone (20 mg/d) in our case, compared to the standard guideline recommendation, was a deliberate clinical decision. This approach was based on the patient’s advanced age and our goal to mitigate the risk of potential corticosteroid-related side effects, such as hyperglycemia and infection, especially while the underlying malignancy was being investigated and treated.^[28]^ In this case, the patient was treated with oral prednisone tablets at 20 mg/d, which showed significant therapeutic efficacy; however, the dosage was less than the guideline dosage. This result suggests that in actual clinical practice, an individualized treatment plan may be more important, and close monitoring of the patient’s condition and response to medication is necessary to ensure treatment safety and efficacy. Attention should also be paid to monitoring the side effects of the medication.

This case emphasizes that CAM may lack specific clinical manifestations, and symmetrical proximal muscle pain may be an early manifestation of CAM, especially in the absence of typical extramuscular symptoms. The detection of MSAs is of great significance for diagnosis, and their sensitivity to hormone therapy provides guidance for treatment. Additionally, this case reminds us of the need to actively search for potential malignancies during the treatment of myalgia to improve the prognosis of patients. Further research is needed to explore the pathogenesis of CAM, search for more specific biomarkers, and develop standardized diagnostic and treatment guidelines to improve the early diagnosis rate and therapeutic efficacy for patients with CAM.

## Author contributions

**Conceptualization:** Xiuqing Huang.

**Data curation:** Jian Xu.

**Formal analysis:** Shucong Peng.

**Methodology:** Xiuqing Huang.

**Resources:** Jian Xu.

**Writing – original draft:** Jian Xu.
